# Syphilis of Larynx

**Published:** 1874-02

**Authors:** Morell Mackenzie

**Affiliations:** London


Syphilis of the Larynx.—*By Morell Mackenzie, M.D., London.* In speaking of laryngeal phthisis and of its different varieties, we remarked how slightly oftentimes the outward and inward configuration of the vocal organ is changed. In tertiary syphilis, on the contrary, enormous changes of shape frequently take place. In these cases, too, the constitution is generally weak, and a certain amount of cachexia exists. When we have alteration of voice, dyspnœa, and difficulty of swallowing, it is often obligatory to make a differential diagnosis between cancer, phthisis, and syphilis of the larynx. Apart from the great differences in form, which occasionally exist, of healthy larynges, we must remark that syphils *always* produces change of form, and in this we find a distinguishing feature of great importance between syphilitic laryngitis and laryngeal phthisis.

In cancer we do not perceive mere alteration of form; displacement is also produced. Again, in laryngeal phthisis we never get ulceration without previous thickening, whereas in syphilis one often finds ulcration without anterior deposit. This is a good help toward a differential diagnosis.

Phthisis and cancer always cause ulceration, but to a limited extent only. In syphilis, ulceration is very extensive. Finally, syphilitic disease often causes great narrowness of the laryngeal opening. It is true that, in simple chronic laryngitis, we likewise get this same narrowness of the larynx, but it exists then without the change of form that occurs in syphilis of the larynx.

In secondary syphilis, great differences in practice are accepted among practitioners. By the majority secondary symptoms are treated with mercury. A few others (and among these Dr. Mackenzie classes himself) do not consider this habit justified by the results. For his own part, Dr. Mackenzie does not believe the administration of salts of mercury ever prevents the appearance of tertiary affections of the throat, and does not, therefore, approve of their use. The solutions of sulphate of copper (generally xv grs.—℥ j) and tincture of iodine being properly employed in local applications, we can generally have a cure in two or three months. Of all local remedies, sulphate of copper is the most efficacious. Sometimes, in very stubborn cases, we may have recourse with advantage to the use of a solution of nitrate of silver (ʒ j of the salt in each fluidounce of water).

In the space of six months to a year these secondary manifestations will wear themselves out spontaneously, for after all syphilis is but an exanthematous affection, and its eruption is to be likened to the eruption of measles or small-pox.

Regarded in this light, the secondary phenomena do not require a constitutional treatment. Not so tertiary syphilis. This form of the disease demands imperatively to be treated in an energetic manner. Iodide of potassium must be given internally (grs. x three times a day) combined with spirits of ammonia, and dissolved in large quantities of water. The iodide does not, administered in this way, produce irritation of the throat, and the quantity of water taken has a tendency to carry off through the emunctories of the economy the very poison itself of the disease.

Those cases of syphilis which occur in persons suffering from tubercular disease of the lungs are extremely intractable, and the physician can rarely if ever be of any use.

